# Single-cell transcriptomics reveals tumor microenvironment remodeling in hepatocellular carcinoma with varying tumor subclonal complexity

**DOI:** 10.3389/fgene.2024.1467682

**Published:** 2024-08-29

**Authors:** Jian Shi, Yanru Zhang, Lixia Xu, Fang Wang

**Affiliations:** ^1^ Department of Oncology, The First Affiliated Hospital, Sun Yat-Sen University, Guangzhou, China; ^2^ Institute of Precision Medicine, The First Affiliated Hospital, Sun Yat-Sen University, Guangzhou, China

**Keywords:** intra-tumoral heterogeneity, tumor microenvironment, subclonal structure, immune activation, cell-cell communications

## Abstract

**Introduction:**

The complexity of tumor cell subclonal structure has been extensively investigated in hepatocellular carcinoma. However, the role of subclonal complexity in reshaping the tumor microenvironment (TME) remains poorly understood.

**Methods:**

We integrated single-cell transcriptome sequencing data from four independent HCC cohorts, involving 30 samples, to decode the associations between tumor subclonal complexity and the TME. We proposed a robust metric to accurately quantify the degree of subclonal complexity for each sample based on discrete copy number variations (CNVs) profiles.

**Results:**

We found that tumor cells in the high-complexity group originated from the cell lineage with FGB overexpression and exhibited high levels of transcription factors associated with poor survival. In contrast, tumor cells in low-complexity patients showed activation of more hallmark signaling pathways, more active cell-cell communications within the TME and a higher immune activation status. Additionally, cytokines signaling activity analysis suggested a link between *HMGB1* expressed by a specific endothelial subtype and T cell proliferation.

**Discussion:**

Our study sheds light on the intricate relationship between the complexity of subclonal structure and the TME, offering novel insights into potential therapeutic targets for HCC.

## Introduction

Hepatocellular carcinoma (HCC) is the third most lethal cancer in the world, with patients often experiencing post-surgical recurrence and metastasis, leading to poor survival outcomes (Wang et al., 2021). Tumor heterogeneity is the main cause of drug resistance and treatment failure (Dagogo-Jack and Shaw, 2018; Marusyk et al., 2020; Vasan et al., 2019), which encompasses the cellular diversity and dynamics both among tumors in different patients and within individual tumor. The tumor heterogeneity is manifested through genomic mutations, aberrations in epigenetic modifications, transcriptional alterations, and changes at the protein level (Sun and Yu, 2015). Additionally, extrinsic factors such as hypoxia, pH levels, and the interactions between tumor cells and other stromal components within the tumor microenvironment (TME) contribute to the diversity and dynamics in tumor genotypes and phenotypes (Sun and Yu, 2015). Previous studies, such as those conducted by TCGA and ICGC, have integrated multi-omics profiles to delineate the heterogeneous molecular features of cancers and explored the subtyping of cancers, guiding personalized therapeutic strategies (Cancer Genome Atlas Research, 2013). Tumor heterogeneity also underpins tumor evolution, as tumor cells continuously accumulate genetic and epigenetic variations, forming heterogeneous subclones which possess different survival fitness and undergo dynamic processes of subclonal selection and elimination under selective pressures such as nutrient availability and metabolism (Davis et al., 2017; Greaves and Maley, 2012). Therefore, understanding the complexity of subclonal structure within tumor is crucial for elucidating tumor biology, disease progression, and response to therapy.

The recent advancements in single-cell sequencing technology (Kashima et al., 2020; Yu et al., 2021) have provided unprecedented insights into the composition, function, and spatial organization of immune and stromal cells within the tumor microenvironment (TME), leading to a deeper understanding of its complexity and diversity. In the process of tumor progression, tumor heterogeneity continuously remodels the tumor microenvironment by altering the transcription levels of target genes in non-malignant cells within the TME, which leads to divergences in developmental trajectories, immune landscapes, and intercellular networks (Wu et al., 2021; Leader et al., 2021; Zhang A. et al., 2022; Voit et al., 2023). Specifically, heterogeneous secretion of cytokines by tumor cells can also modulate immune-tumor interactions, as demonstrated by studies in mouse models (Liu et al., 2013; Knoche et al., 2021). Tumor cells can induce profound phenotypic changes in non-immune stromal components within the TME, impacting the immune components and their activities. For instance, oncogenic BRAFV600E signaling in human melanoma cells has been shown to interfere with T cell-mediated anti-tumor responses by modulating the phenotype of cancer-associated fibroblasts (Khalili et al., 2012; Binnewies et al., 2018). Furthermore, immune cells within the microenvironment can eliminate clones with high mutational burdens (high immunogenicity) through the process of immune editing, maintaining a balance that ultimately favors the selection of tumor variants capable of evading immune surveillance (Tsai et al., 2023; Polyak et al., 2009; Nam et al., 2021; Dunn et al., 2002; O’Donnell et al., 2019). Thus, the genotype and phenotype of tumor cells and the TME are inextricably linked. However, the intricacies of their interactions and how the subclonal complexity reshapes the TME remain largely unexplored.

In this study, we performed an integrated single-cell analysis of HCC patients with untreated primary tumors. We developed a metric to quantify the tumor subclonal complexity based on Shannon entropy theory. Our investigation focused on systematically elucidating the relationship between the subclonal complexity and cancer cell state, the functional activity of the immune cells. We found that cell-cell interaction landscape and TME polarization varied with subclonal complexity. Our findings underscore the significance of subclonal complexity in modulating the tumor microenvironment and its implications for immune responses.

## Materials and methods

### scRNA-seq datasets

The datasets of HCC were acquired from GEO, including 4 scRNA-seq cohorts (GSE156625 (24), GSE149614 (25), GSE151530 (26), GSE189903 (27)). Specifically, GSE156625 (https://www.ncbi.nlm.nih.gov/Traces/study/?acc=PRJNA658541&o=acc_s%3Aa) was the raw sequencing data and the other 3 datasets are processed read count matrices. To accurately analyze the impact of tumor subclonal complexity on the remodeling of the tumor microenvironment, we established the following criteria for sample selection: 1) all samples are from the 10X Genomics platform; 2) availability of raw sequencing data or read count expression matrices; 3) samples are untreated; 4) samples are primary tumors without invasion, recurrence and metastasis; 5) for multi-region sequencing data, we selected samples from the tumor core region whenever possible. Finally, only 30 samples were retained for further analysis (Supplementary Table S1).

### Pre-processing of scRNA-seq data

Raw reads from GSE156625 were processed to generate gene expression matrices by using the standard internal pipeline based on the Cell Ranger toolkit (3.1.1). Then the expression matrices of each individual sample were converted to Seurat object by the “CreateSeuratObject” function from Seurat package (4.3.0) (Hao et al., 2021). We firstly filtered out cells that had either lower than 200 or higher than 8,000 expressed genes. Furthermore, the cells with the percent of mitochondrial genes over 40% of total expressed genes were discarded. Additionally, we initially removed doublet cells by DoubletFinder (2.0.3) (McGinnis et al., 2019) to avoid the potential influence on analytical results. A total of 67,125 cells were retained for further analysis.

### Dimensionality reduction, clustering, DEGs identification, and major cell type annotation

To better reflect the biological features from the data, we used the “NormalizeData” function to normalize the expression matrices for the Seurat object. And the top 2,000 highly variable genes (HVGs) identified by “FindVariableFeatures” function were used to scale data through “ScaleData” function. The principal component analysis (PCA) was carried out by “RunPCA” function to generate 50 PCs, followed by “RunHarmony” function implanted in Harmony package (0.1.1) (Korsunsky et al., 2019) with the top 30 PCs to eliminate batch effect. The clustering analysis was implemented depended on the integrated joint embedding produced by Harmony with the Louvain algorithm after computing a shared nearest-neighbor (SNN) graph. The UMAP technique was used to create a 2D map on which the discovered clusters were displayed. Then we used “FindAllMarkers” function to identify over-expressed genes in specific cluster when compared with the other clusters (adjusted *P*-value <0.05, only.pos = T and logfc.threshold = 0.25). The well-known cell markers (Ma et al., 2021; Liu et al., 2023) were used to annotate the clusters: Endothelial (*ENG*, *VWF*, *PECAM1*), Fibroblasts (*ACTA2*, *COL1A2*, *PDGFRB*), T cells (*CD2*, *CD3D*, *CD3E*, *CD3G*), B cells (*CD79A*, *CD79B*, *MS4A1*, *BANK1*), Myeloid (*CD14*, *CD68*), Plasma (*MZB1*, *DERL3*, *SDC1*), Hepatocytes (*APOA2*, *ALB*, *APOA1*, *AMBP*), proliferative T (*TOP2A*, *MKI67*, *TUBB*).

### Cell subtype annotation

We further clustered T cells, endothelial cells and myeloid cells following a similar pipeline as described above, including normalization, highly variable genes identification, dimensionality reduction, batch effect correction with Harmony and clustering. Within each cell subtype, an iterative procedure that a cell cluster displaying at least two canonical marker expression of 7 major cell type was determined as doublet cells to be removed, if any, and reclustered the remaining cells, was adopted to ensure the reliability of the study. Specifically, we used the following well-known markers (Cheng et al., 2021; Zheng et al., 2021) for subtype identification. NK (*GNLY*, *KLRF1*, *NKG7*), CD4+_CCR7 (*CD4*, *CCR7*), CD4+_IFNG+ (*CD4*, *IFNG*), CD4+_FOXP3_Treg (*CD4*, *FOXP3*, *IL2RA*), CD8+_GZMK (*CD8A*, *GZMK*), CD8+_TNFSF9 (*CD8A*, *TNFSF9*), macrophage (*C1QC*, *APOE*, *CD68*), monocyte (*FCN1*, *LYZ*), cDC1_CLEC9A (*CLEC9A*, *FLT3*), cDC2_CD1C (*CD1C*, *FCER1A*), cDC3_LAMP3 (*LAMP3*, *CCR7*), mast (*KIT*). Similarly, endothelial cell were also confirmed by canonical markers (Goveia et al., 2020; Schupp et al., 2021), such as activated_PCV (*ACKR1, SELP, VCAM1, POSTN*), Capillaries_EDNRB (*EDNRB, CA4, HPGD, IL1RL1*), Capillaries_FCN3 (*FCN3, BTNL9, NOSTRIN, EDN*), arteries_GJA5 (*GJA5, EFNB2, SOX17, DKK2*), tip_CXCR4 (*CXCR4, ADM, ANGPT2, APLN*), RBP4+_EC (*RBP4, IGFBP7*), CXCL10+_EC (*CXCL10*, *GBP1, SOD2*). To accurately annotate proliferative T cells, we calculated the averaged Pearson correlation coefficient and averaged *p*-value for each proliferative T cell against other T cell subtypes and NK subtypes based on single-cell expression profiles. Proliferative T cells were excluded if the mean correlation coefficients were all less than 0.4 or the mean *p*-values all exceeded 0.05. Otherwise, they were assigned to the subtype with the highest average correlation.

### CNVs estimation and malignant cells identification

We inferred copy number variations (CNVs) for each cell through the InferCNV (1.10.1) package of R. The non-malignant cells were applied as the reference to estimate the CNVs values of the hepatocytes (observation). The genes were sorted by their genomic locations on each chromosome. Specifically, The InferCNV analysis was implemented with parameters “denoise = T, HMM = T, analysis_mode = “subclusters”, cutoff = 0.1, cluster_by_groups = T”.

To identify the malignant cells from the hepatocytes, we designed the CNVs score quantifying the degree of CNVs fluctuation for each cell as follows:

$${CNV}_{score}=\sqrt{\sum_{i=1}{\left(X_{i}-X_{\text{mean}}\right)}^{2}}\;/\;n$$

where $X_{i}$ represented the CNVs value of gene *i* in the cell, $X_{\text{mean}}$ was the mean CNVs value of gene *i* in the cell, and n was the number of the genes in the cell. If the CNVs score of a hepatocyte exceeded the threshold determined by the intersection of the distributions of CNVs score in hepatocytes and non-malignant cells, then the hepatocyte was considered malignant (Supplementary Figure S1C).

### Identification of intratumor NMF programs

**FIGURE 1:**
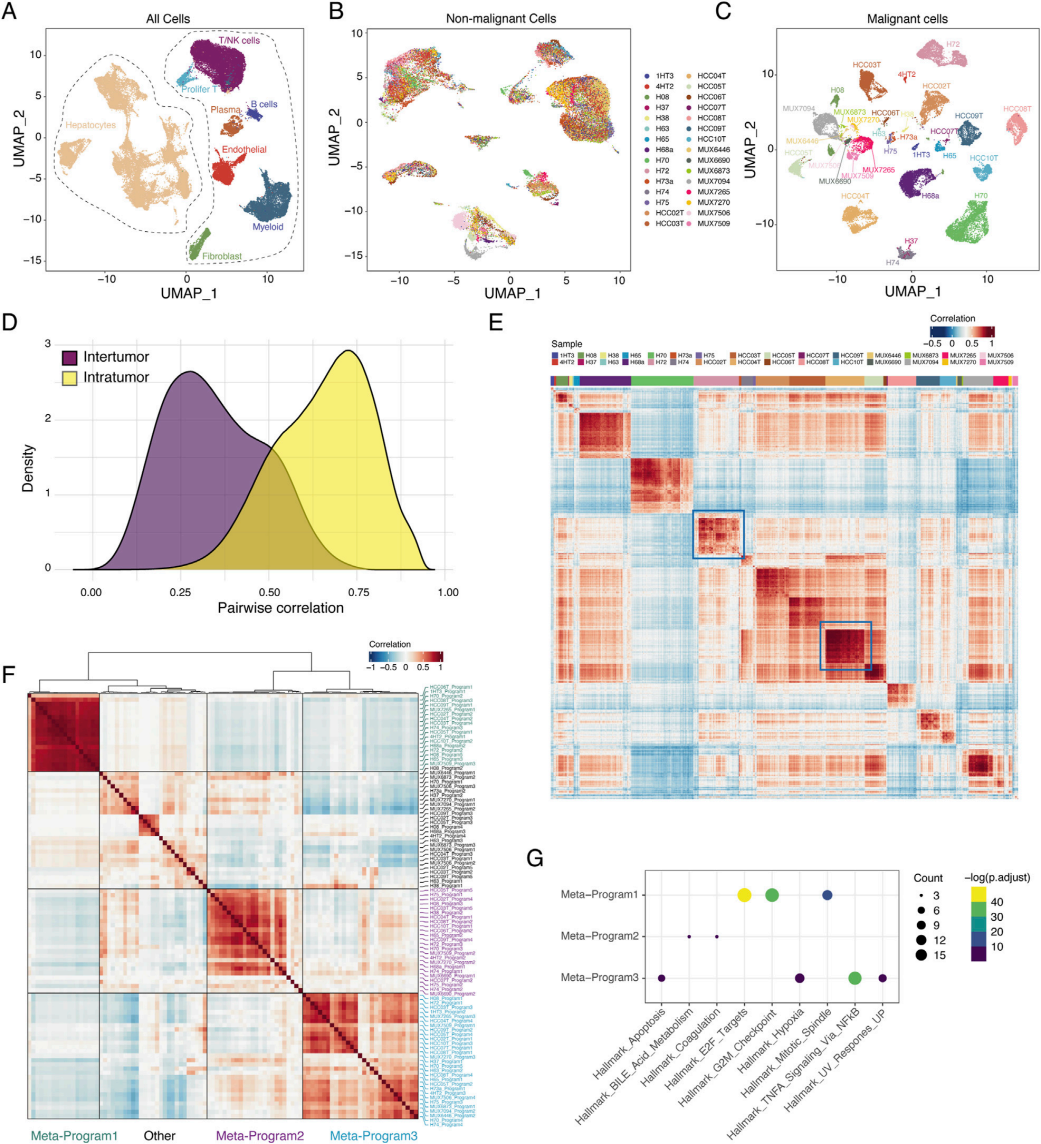
Tumor heterogeneity landscape of HCC. **(A)** UMAP plot of all 66609 cells from 30 samples. Cells were annotated based on the well-known markers. **(B)** UMAP plot of 30955 malignant cells from 30 samples. Cells were colored by samples. **(C)** UMAP plot of 34777 non-malignant cells from 30 samples. Cells were colored by sample. **(D)** Distributions of pairwise correlation within intra-tumor and inter-tumor. **(E)** Pairwise correlation of all malignant cells from 30 samples. Red represents positive correlation and blue represents negative correlation. **(F)** Heatmap of average correlations across 30 samples between pairs of expression programs. **(G)** Dot plot displaying significantly enriched pathways for genes of each meta-program. The size of the dot represented the number of genes in the indicated hallmark (the larger, the more genes included) and the color shade of the dot indicated the *P* value after FDR correction (the yellower, the smaller adjusted *P* value).

The non-negative Matrix Factorization (NMF) algorithm was applied to identify the underlying expression programs from the malignant cells based on the NMF R package. We employed NMF (rank = 2:6, nrun = 10) to the relative expression matrices of the top 2000 HVGs in each sample with all negative values converted to 0. We chose the optimal *rank* value at which the cophenetic coefficient started producing the maximum descent (Supplementary Figure S1E). In total, we identified 98 programs from 30 samples (Supplementary Table S2). The 30 genes of each expression program with the top NMF scores were input into “AddModuleScore” function to generate the program score for malignant cells in each sample. We calculated the correlations between the 98 program scores in each sample individually. After excluding clustering groups containing less than half of samples, we finally extracted 3 meta-programs from the 98 programs according to hierarchical clustering of averaged correlations of pairs of programs across all samples (Figure 1F; Supplementary Table S3). Each meta-program, retaining 30 genes with the highest average NMF score, was used to perform pathway enrichment analysis.

### CNV complexity score based on single cell CNVs profiles

Shannon entropy was used to quantify the CNV complexity for each sample based on discrete CNVs profiles inferred from InferCNV. Firstly, we calculated the frequency of copy number gains and losses for each gene within the tumor as follows:

$$LVF=n_{loss}/n$$

$$GVF=n_{gain}/n$$

where n represented the total number of malignant cells, $n_{loss}$ was the number of cells in which the copy number of the gene was lost, and $n_{gain}$ was the number of cells in which the copy number of the gene was gained. Specifically, Discrete CNVs value of the gene greater than (less than) 3 was considered to be gained (lost). The frequency of copy number gain and loss were divided into 10 equal-length frequency intervals separately (i.e., Loss:0–0.1, 0.1–0.2, …, 0.9–1; Gain:0–0.1, 0.1–0.2, …, 0.9–1). After mapping all the genes to equal-length frequency intervals according to the LVF and GVF of the genes, we then counted the number of genes belonging to each equal-length frequency interval and calculated the frequency of the number of genes in each interval. Finally, the CNV complexity score could be calculated for each sample as follow:

$$CNV\_complexity\_score=-\sum_{i}P\left(X_{i}\right)\log_{2}P\left(X_{i}\right)$$

where i was the *i*th interval, ${\;X}_{i}$ was the number of genes falling into the *i*th interval, and $P\left(X_{i}\right)$ was the frequency of the number of genes in the *i*th interval.

### Simulation data and method evaluation

To evaluate whether the CNV complexity score could accurately distinguish between polyclonal and monoclonal samples, we used computer simulations to generate single-cell CNV profiles for 100 polyclonal samples and 100 monoclonal samples as the ground truth data, respectively. Briefly, the size of cell population N ∼ U [200, 2000] and the total number of clones C ∼ U [5, 20] were generated for each sample. For each clone, the size of clone was generated through binomial distribution B (N, *p*). For monoclonal samples, we assumed that *p* = 0.9 for one dominant clone and *p* = 0.1/(c-1) for other rare clones. We considered int (N/2) subclones in the tumor cells, where int takes the nearest integer in the polyclonal samples. We used *p* = 2/N to generate the size of subclone. For detailed steps of the entire simulation data generation process, please refer to Wang et al. (2023). We then applied the three methods (CNV complexity score, Guo et al.’s and Ma et al.’s) to the simulated single-cell CNV profiles which had been labeled as either monoclonal or polyclonal to calculate scores. A higher score for this sample means that the algorithm is more likely to predict the sample as a polyclonal sample. Finally, we used R package pROC (Robin et al., 2011) to calculate precision, recall, and the area under the receiver operating characteristic curve (AUC).

### Gene set enrichment analysis, pathway enrichment analysis and ssGSEA

Gene set enrichment analysis (GSEA) and pathway enrichment analysis were performed using R package clusterProfiler (Yu et al., 2012). The ssGSEA algorithm was used to calculate the activity score of the gene sets for each cell based on GSVA package (Hanzelmann et al., 2013). We obtained 13 cancer functional states from CancerSEA (Yuan et al., 2019), as well as a collection of 50 cancer Hallmark gene sets downloaded from MsigDB (Liberzon et al., 2015). The signature gene lists of macrophages (e.g. Angiogenesis, Phagocytosis, MHC I molecules, MHC II molecules; Supplementary Table S5) and T cells (e.g. Cytotoxic, Exhaustion, Terminally exhaustion, Proliferation, TCR signaling pathway, Type II interferon response; Supplementary Table S6) were collected from previous studies (Rooney et al., 2015; Long et al., 2022; Li et al., 2020; Jiang et al., 2021; Zhang Y. et al., 2022).

### CytoTRACE and pseudotime analysis

The R package CytoTRACE v.0.3.3 (47) was applied to predict the differentiation state of cells from the single-cell RNA-seq (scRNA-seq) profiles. The CytoTRACE score range from 0 to 1, with higher scores being associated with greater stemness (less differentiation) and *vice versa*, which help us determine the starting point of differentiation. We employed Monocle2 (Qiu et al., 2017) to discover the differentiation trajectory for T cells and endothelial cells. The top 2,000 DEGs between the cell subgroups were used to create the DDRTree. Utilizing the root state determined by the CytoTRACE, each cell was given a pseudotime value using the “order_cells” function. The cells were ordered and visualized with the “plot_cell_trajectory” function.

### TF regulatory network construction and critical TFs identification

The SCENIC (Aibar et al., 2017) was used to construct TF regulatory network for malignant cells in different subclonal complexity. Briefly, GRNBoost2 estimated the co-expression network and RcisTarget was used to identify the regulons. The regulon activity was then measured by AUCell for every cell. The differently activated TFs regulons between different subclonal complexity were identified by wilcox.test(). The TF-gene regulatory network was visualized by Cytoscape (Shannon et al., 2003).

### Evaluation of associations between critical TFs and survival outcomes

To explore the prognostic power of TFs, we downloaded two gene expression datasets from cBioPortal (Gao et al., 2013) and GEO (GSE76427) for HCC patients, which contained clinical information. HCC patients from TCGA were treated as a training dataset, and HCC patients from GSE76427 were used as an independent validation dataset. A univariate Cox proportional hazards regression analysis was performed to evaluate the association between the critical TFs and patients’ OS. Only TFs with *p*-value <0.05 were selected to further conduct variable selection according to stepwise Cox proportional hazards regression analysis. Finally, we created a risk-score formula based on TFs expression weighted by Cox regression coefficients.

$$Risk\;score=\sum_{i}^{N}\left({coef}_{i}\times {expr}_{i}\right)$$

where N was the number of selected TFs, ${\text{expr}}_{i}$ was the expression value of the *i*th gene, and ${\text{coef}}_{i}$ was the Cox regression coefficient of the *i*th gene in the univariate Cox regression analysis. Patients were divided into high-risk and low-risk group based on the median risk score. KM curves were performed to compare the OS and DFS between two groups. To evaluate whether risk score could be an independent prognostic factor, Multivariate Cox proportional hazards regression model was conducted with risk score, age, sex, height, weight, race as covariates.

### Cell-cell communications analysis

We applied CellPhoneDB (Efremova et al., 2020) to infer cell-cell communication between different cell types. The specific ligand-receptor interactions between cell types were identified based on permutation test. The interaction score represented the total mean of the individual ligand-receptor partner average expression value in the corresponding interacting pairs of cell types. To predict ligands driving the transcriptomic changes of target cells, we further performed NicheNet analysis (Browaeys et al., 2020) between RBP4+_EC and T cells with the “nichenet_seuratobj_aggregate” function. Specifically, the differentially expressed genes (DEGs) among T cells between different subclonal complexity with high complexity group as control were used as gene sets of interest and all expressed genes in T cells were used as background of genes.

### Cytokines signaling activity calculation

We used CytoSig algorithm (Jiang et al., 2021) to quantify the signaling activity of cytokines for T cells. The CytoSig was a linear model predicting cytokines signaling activity score for each cell and covered 51 cytokines provided by the curated training data. We implemented CytoSig for single-cell transcriptional profiles of T cells in Python 3.

### Statistical analysis

Continuous variables were compared by the Wilcoxon rank-sum test and categorical variables were compared by the chi-squared test. Pearson correlation was calculated by cor () and cor. test (). The log-rank test was used to compare Kaplan–Meier curves. When *p* < 0.05, the differences were considered to be significant. All statistical analyses were conducted using R software version 4.1.2 (http://www.rproject.org).

## Results

### The transcriptional diversity analysis reveals transcriptional programs in primary HCC

We downloaded 30 untreated primary HCC samples from four cohorts of single-cell RNA-seq data (Sharma et al., 2020; Lu et al., 2022; Ma et al., 2021; Ma et al., 2022) (Supplementary Table S1). After quality control, we obtained a total of 66,609 high-quality cells with a median of 2,220 cells per sample. Cell types were annotated by canonical cell markers, identifying 31,832 hepatocytes and 34,777 non-malignant cells (Figure 1A; Supplementary Figure S1A). We inferred copy number variations (CNVs) based on scRNA-seq data using InferCNV((Project. iotTC)) (Supplementary Figure S1B). Malignant and non-malignant hepatocytes were distinguished by their accumulated CNV scores (see Section Methods, Supplementary Figure S1C), ultimately identifying 30,955 malignant cells.

We performed clustering and UMAP visualization separately for malignant and non-malignant cells. Consistent with previous findings, non-malignant cells exhibited distinct separation by cell type but showed a mixture of different samples (Supplementary Figure S1D; Figure 1B), whereas malignant cells exhibited high inter-tumor heterogeneity (Figure 1C) (Puram et al., 2017). The transcriptomic similarity among inter-tumor cells was lower than that among intra-tumor cells (Figure 1D). However, the similarity of intra-tumor malignant cells varied across patients; for instance, HCC04T showed relatively high similarity, while H72 exhibited lower similarity among malignant cells (Figure 1E).

To identify underlying transcriptional programs, we applied non-negative matrix factorization (NMF) (Gaujoux and Seoighe, 2010) to each sample. The identified gene programs tend to be mutually exclusive among malignant cells within sample, and were negatively correlated (Supplementary Figure S1E–G). A total of 98 gene programs were identified across the 30 tumor samples (Supplementary Table S2). We then performed hierarchical clustering on these 98 expression programs, extracting three meta-programs (Figure 1F; Supplementary Table S3) that encompassed more than half of the samples. These three meta-programs were associated with distinct signaling pathways: Meta-program 1 was enriched in cell cycle-related pathways, Meta-program 2 was enriched in metabolic pathways, and Meta-program 3 was enriched in stress response-related pathways (Figure 1G).

### Quantifying intra-tumor subclonal complexity using CNV profiles

**FIGURE 2:**
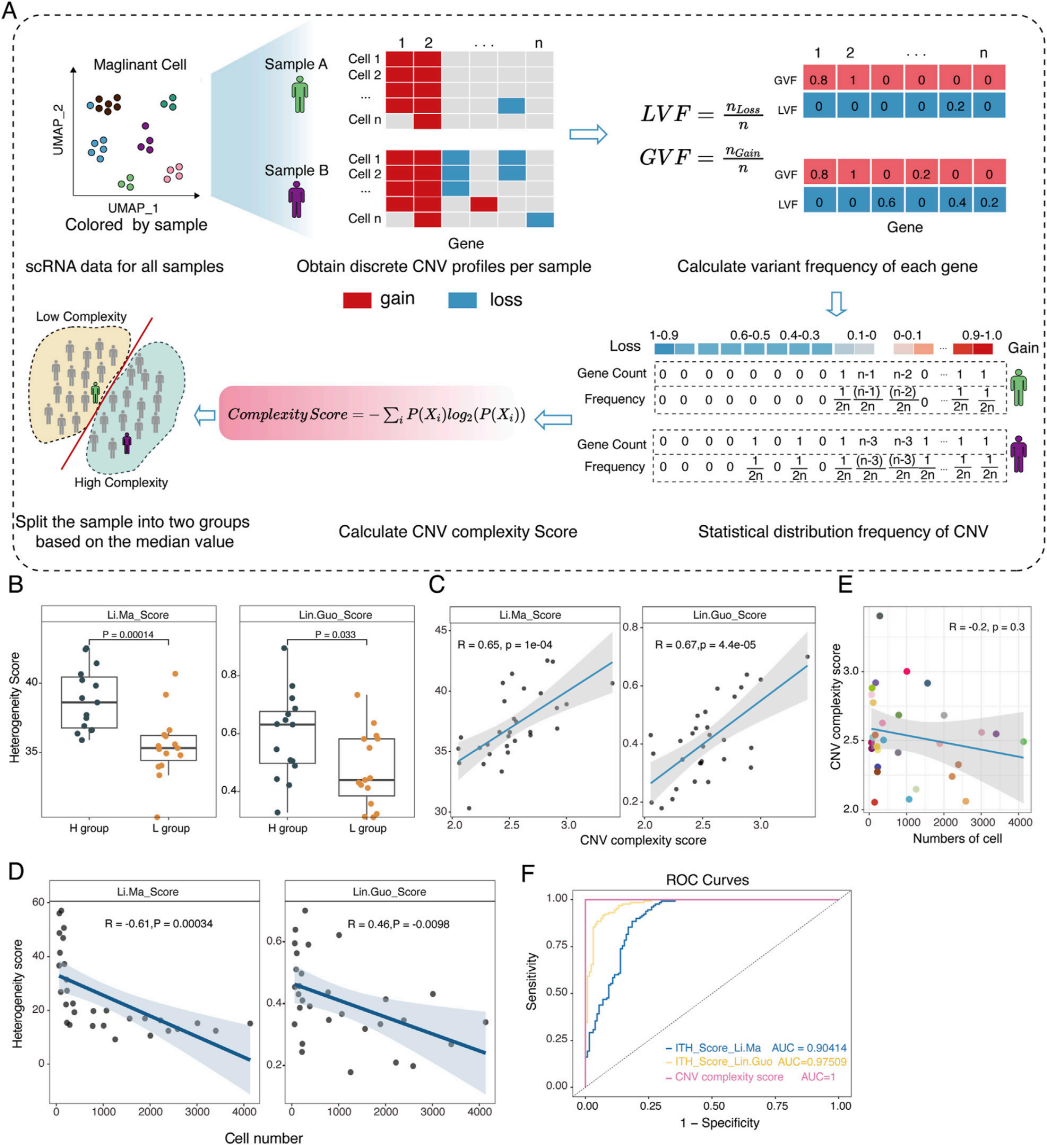
Construction and validation of the robust CNV complexity score. **(A)** Overview of the novel scoring strategy based on Shannon entropy theory. **(B)** Comparative analysis of our method and two other strategies (Ma et al. and Guo et al.) in the real discrete CNV profiles inferred by InferCNV. **(C)** Correlation analysis of CNV complexity score with Ma et al.’s score (left) and Guo et al.’s score (right) respectively. **(D)** Scatterplot showing the correlation between the number of cells and Ma et al.’s score (left), Guo et al.’s score (right). Each dot represents a sample. **(E)** Scatterplot showing the correlation between the number of cells and the CNV complexity score. Each dot represents a sample. **(F)** ROC curve of CNV complexity score, Ma et al.’s score and Guo et al.’s score in the simulated datasets.

To explore intra-tumor subclonal complexity, we proposed a metric to quantify CNV complexity based on the discrete CNVs profiles inferred from InferCNV (Figure 2A, method). We quantified CNV complexity for each HCC sample and classified samples into high (H) and low (L) groups based on the median CNV complexity score. We found that CNV complexity scores were significantly correlated with two other heterogeneity scores proposed by (Ma et al., 2019) and (Guo et al., 2022) respectively (Figures 2B, C). Moreover, the CNV complexity score was more robust than the other two heterogeneity scores, both of which were negatively correlated with the number of the cells (Figures 2D, E). Simulation data further demonstrated the robustness of the CNV complexity score (Supplementary Figure S2A).

Finally, we evaluated the three methods on simulated datasets generated *in silico* (Supplementary Figure S2B, C). The CNV complexity score showed excellent performance in reflecting the degree of subclonal complexity, as demonstrated by a receiver operating characteristic (ROC) curve with the highest AUC value, which was superior to the other methods (Figure 2F). Overall, our constructed CNV complexity score demonstrates excellent stability and sensitivity, and more importantly, it is not influenced by the number of cells, providing a more accurate quantification of subclonal complexity in samples.

### Phenotypic regulatory plasticity in HCC samples with varying subclonal complexity

**FIGURE 3:**
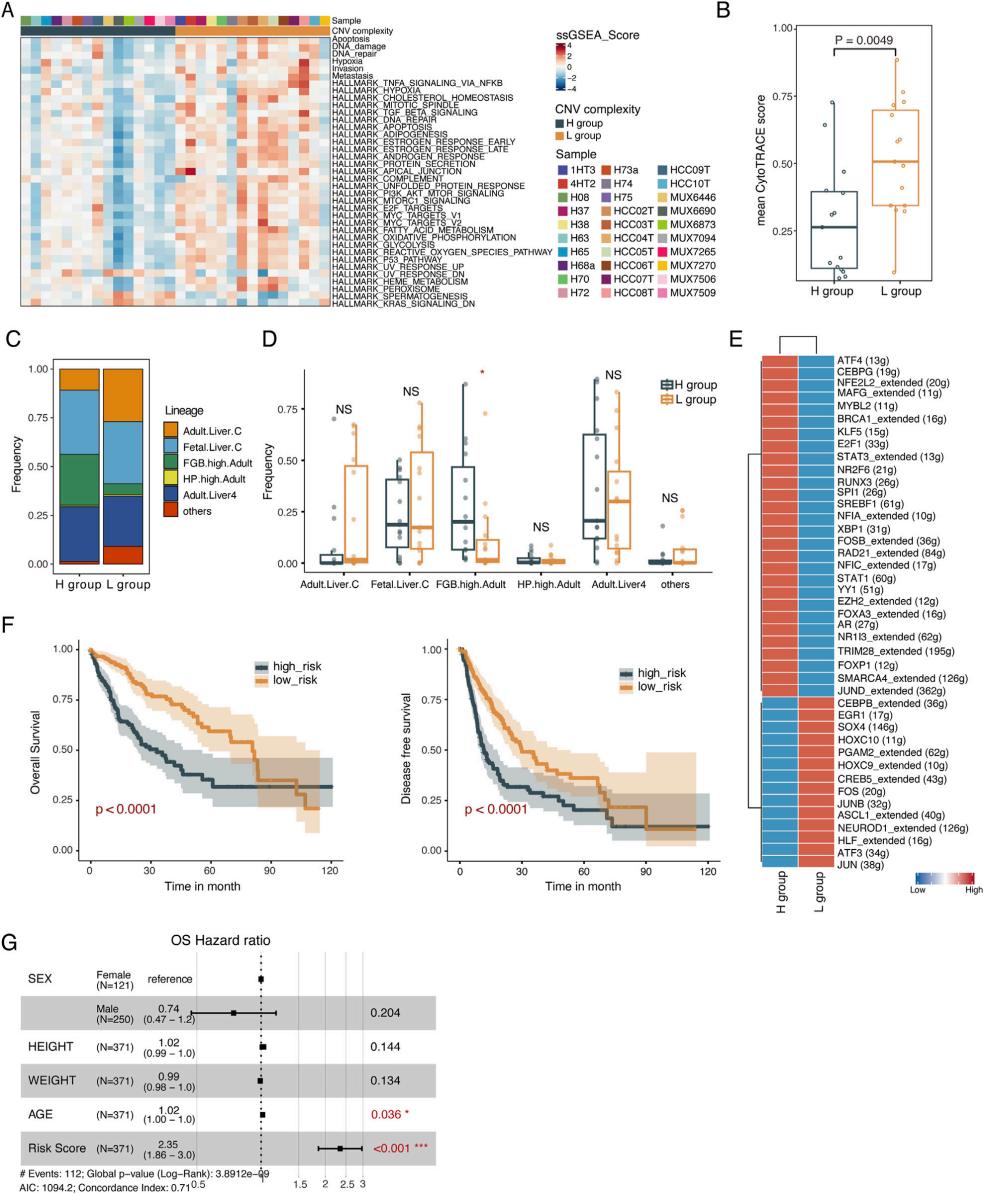
Identification of subclonal complexity-specific TFs. **(A)** Heatmap of the ssGSEA score of 37 cancer functional states significantly differed between L and H groups. Two-sided Wilcoxon rank-sum test. **(B)** Boxplot showing the CytoTRACE score of the L and H groups. The CytoTRACE score for each sample was the averaged CytoTRACE score of malignant cells from the sample. Two-sided Wilcoxon rank-sum test. **(C)** Histogram indicating the proportion of cells lineages in malignant cells from L and H groups. Chi-squared test. **(D)** Boxplot showing the fraction of cell lineages in L and H groups. *p < 0.05; **p < 0.01; ***p < 0.001; NS not significant. **(E)** Heatmap showing the TF activity for malignant cells derived from L and H groups. **(F)** Kaplan-Meier plots of risk score on OS (left) and DFS (right). **(G)** Forest plot showing the HR (95% CI) for OS. The multivariate Cox proportional hazard models. HR, hazard ratios; CI, confidence interval.

To explore the phenotypic plasticity underlying different subclonal complexity, we used cancer functional gene sets (Supplementary Table S4) along with ssGSEA and identified 37 gene sets with significant differences between the H and L groups, with 34 functional gene sets being significantly upregulated in the L group (*p* < 0.05, Wilcoxon rank-sum test, Figure 3A; Supplementary Figure S3). For instance, the L group was significantly enriched for hypoxia, invasiveness, metastasis, emphasizing varying signaling activity between the H group and L group. Malignant cells are susceptible to epithelial-mesenchymal transition under hypoxic conditions, gaining invasive and metastatic abilities, accompanied by a transition from low to high stemness. Consistent with hypoxia, invasion and metastasis activities mentioned above, it was discovered that the stemness of malignant cells in L group was also significantly higher than that in the H group (*p* = 0.0049, Wilcoxon rank-sum test, Figure 3B) (Gulati et al., 2020), which highlights that stemness of malignant cells is closely related to hypoxia, invasiveness and metastasis.

We further investigated the cell lineage origin of malignant cells using scHCL(Han et al., 2020), and found significantly different distributions of cell lineages between the H and L group (*p* < 0.05, chi-squared test, Figure 3C), with a significantly higher proportion of cell lineage overexpressing FGB in the H group (*p* < 0.05, Wilcoxon rank-sum test, Figure 3D). FGB was significantly upregulated in malignant cells from the H group (*p* < 2.22e-16, Wilcoxon rank-sum test, Supplementary Figure S4A). Altered expression of this gene leads to several disorders, including afibrinogenemia, dysfibrinogenemia, and thrombotic tendency. In summary, malignant cells from samples with varying CNV complexity might undergo diverse transcriptional reprogramming, resulting in different cancer functional states and cell lineage origins.

To investigate the regulatory mechanisms contributing to the different cell lineages and functional phenotypes of tumor cells between the H and L group, we employed SCENIC to identify transcription factors (TFs) regulatory modules (Figure 3E). We then constructed TFs regulatory networks corresponding to the H and L groups using differentially expressed and differential CNV genes targeted by identified TFs (Supplementary Figure S4B; Supplementary Figures S5A, B). Functional enrichment analysis of the TF regulatory network revealed that the upregulated genes in the L group’s TFs regulatory network were enriched in pathways such as hypoxia, apoptosis, TNFA signaling via NFkB and regulation of Wnt signaling. In contrast, the upregulated genes in the H group were involved in pathways regulating epithelial cell proliferation and metabolism-associated pathways (Supplementary Figure S4C).

Among these TFs, we identified four transcription factors *MAFG, YY1, EZH2, AR,* associated with overall survival (OS) which were upregulated in the H group. Samples were stratified into high-risk and low-risk groups based on the median risk score quantified by these four TFs. Kaplan-Meier curve analysis demonstrated poorer survival in the high-risk group compared to the low-risk group in terms of OS and disease-free survival (DFS) (OS: *p* < 0.0001, DFS: *p* < 0.0001, log-rank test, Figure 3F). Further multivariate Cox proportional hazards regression analysis confirmed the independent prognostic value of the risk score (Figure 3G; Supplementary Figure S4D). Finally, we validated the prognostic power of the 4-TF signatures in an external validation dataset (GSE76427). The KM curves of the two groups were significantly different (*p* = 0.0016, log-rank test, Supplementary Figure S4E) and the risk score showed the independent prognostic value (Supplementary Figure S4F).

### Characterizing cell-cell communications in the tumor microenvironment across different subclonal complexity

**FIGURE 4:**
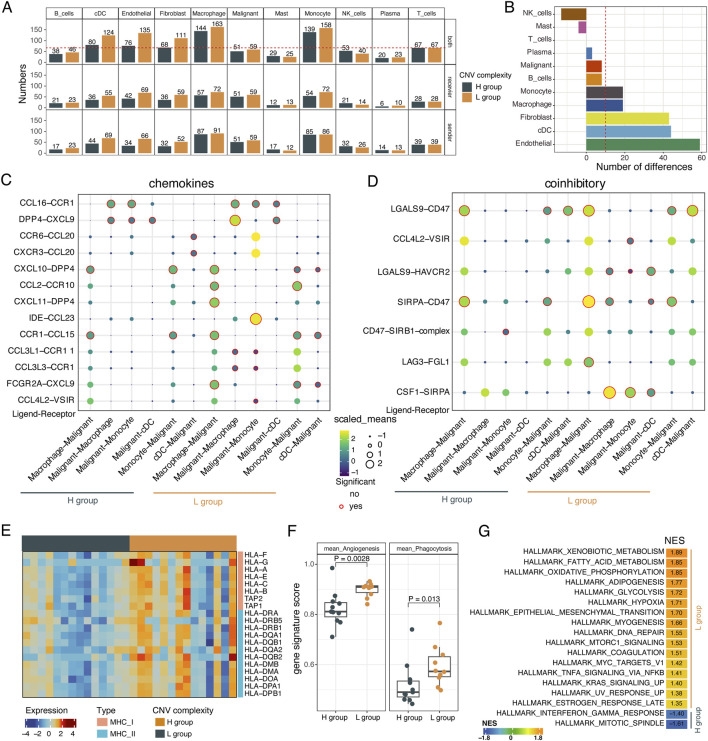
The interactions between malignant cells and non-malignant cells. **(A)** Barplot showing the number of ligand–receptor interactions between malignant cells and other cells within the TME in the L and H groups. Recevier: malignant cells are recevier cells. Sender: malignant cells are sender cells. **(B)** Barplot showing the differences in the number of pairs of ligand-receptor interactions between the L and H groups. **(C)** The chemokine-mediated ligand-receptor interactions between malignant cells and myeloid cells in L and H groups. **(D)** The coinhibitory-mediated ligand-receptor interactions between malignant cells and myeloid cells in L and H groups. **(E)** Heatmap showing the expression levels of MHC I molecules, MHC II molecules from macrophage cells in the L and H groups. Two-sided Wilcoxon rank-sum test. **(F)** Boxplot showing the activity score of angiogenesis and phagocytosis of macrophages cells in L and H groups. Two-sided Wilcoxon rank-sum test. **(G)** Gene set enrichment analysis for macrophage cells. Normalized enrichment score (NES) was used to display the enrichment of the related pathways. P. adjusted <0.05 was considered to be significant.

To investigate the characteristics of cell-cell communications within the tumor microenvironment under different complexity of subclonal structure, we performed cell-cell interaction analysis using CellPhoneDB (Efremova et al., 2020) between malignant cells and non-malignant cells in the H and L groups, respectively. We found that fibroblast and endothelial cells strongly interacted with tumor cells in the L group rather than the H group (Figure 4A). Additionally, myeloid cells, including macrophage, monocytes and dendritic cells (Supplementary Figure S6A, B), showed strong interactions with tumor cells in both the H and L groups, with increased communications observed in the L group (Figure 4B).

We further explored the ligand-receptor interactions between tumor cells and myeloid cells in the L group and H group. We found that many chemokine-mediated interactions were specifically present in the L group, such as the *CCL4L2-VSIR*, *FCGR2A-CXCL9, CCL3L3-CCR1*, *CCL3L1-CCR11*, *IDE-CCL23*, and *CXCL11-DPP4*, *CCL2-CCR10* pathways which suggests that tumor cells in the L group might recruit more myeloid cells. In the H group, there were also a few specific interactions, such as the interactions between the dendritic cell-expressed ligands *CCR6* and *CXCR3* with the tumor cells-expressed receptor *CCL20* (Figure 4C). Additionally, we confirmed that coinhibitory interactions mediated by pathways like *CSF1-SIRPA*, *LAG3-FGL1*, *CCL4L2-VSIR*, and *LGALS9-HAVCR2* were specifically activated in the L group (Figure 4D). Similar results were also observed in the costimulatory-mediated interactions between malignant cells and myeloid cells (Supplementary Figure S6C). Interestingly, the L group was enriched for both coinhibitor- and costimulator-mediated ligand-receptor interaction pairs. In summary, these results revealed that varying subclonal complexity could alter the cell-cell interactions landscape.

Given the differential interaction strength of myeloid cells and tumor cells between the H and L groups, we investigated the functional characteristics of myeloid cells. Myeloid cells from the L group showed upregulation of MHC I and MHC II molecules and higher angiogenesis and phagocytosis signature scores (*p* < 0.05, Wilcoxon rank-sum test, Supplementary Table S5; Figures 4E, F). Based on gene set enrichment analysis (GSEA) of the differentially expressed genes identified in myeloid cells between the L and H groups (Supplementary Figure S6D), more hallmark signaling pathways were enriched in myeloid cells from the L group, including hypoxia, EMT, TNFA signaling via NFkB, DNA repair, apoptosis, and angiogenesis (Figure 4G; Supplementary Figure S6E, F). These results were consistent with what we observed in tumor cells from samples with different complexity of subclonal structure.

### Differential activation and functional states of T Cells with varying subclonal complexity

**FIGURE 5:**
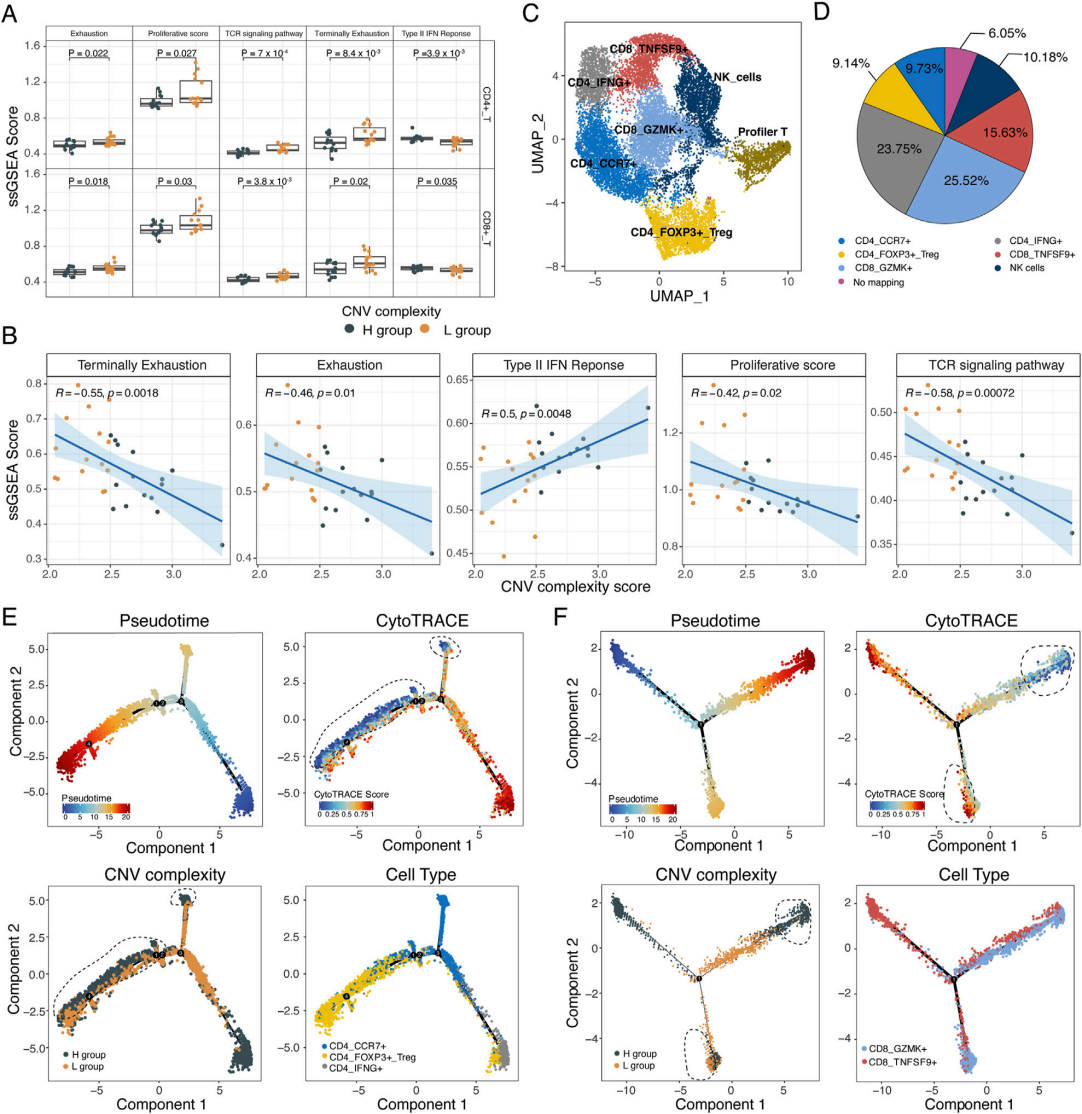
The immune activation of T cells in low CNV complexity. **(A)** Boxplot showing the ssGSEA score of exhaustion, proliferation, TCR signaling pathway, terminally exhaustion, type II IFN response of CD4^+^ and CD8^+^ T cells in the L and H groups. Two-sided wilcoxon rank-sum test. **(B)** Scatterplot showing the correlation between CNV complexity score and the ssGSEA score of exhaustion, proliferation, TCR signaling pathway, terminally exhaustion, and type II IFN response. Each dot represents a sample. **(C)** UMAP plot of 7 T/NK cell subtypes from 30 samples. **(D)** Pie chart showing the annotation results of the proliferative T cells. **(E)** Single-cell trajectory analysis of CD4^+^ T cells. Cells were colored by pseudotime (upper left). Cells were colored by CytoTRACE score (upper right). Cells were colored by subclonal complexity (lower left). Cells were colored by cell types (lower right). Black dashed lines mark cells with both low stemness and high subclonal complexity. **(F)** Single-cell trajectory analysis of CD8^+^ T cells. Cells were colored by pseudotime (upper left). Cells were colored by CytoTRACE score (upper right). Cells were colored by subclonal complexity (lower left). Cells were colored by cell type (lower right). Black dashed lines mark cells with both low stemness and high subclonal complexity, as well as cells with both high stemness and low subclonal complexity.

Although there was no significant difference in tumor-T cells interaction strength between the H and L groups, T cells exhibited significantly different activation statuses based on ssGSEA analysis of functional gene sets (Supplementary Table S6). Both CD4^+^ and CD8^+^ T cells from the L group had higher scores for TCR signaling pathway, exhaustion and proliferation but lower type II interferon response score (*p* < 0.05, Wilcoxon rank-sum test, Figure 5A). Moreover, cytotoxicity-related genes (e.g., *GZMA*, *GZMB*, *GZMH*, *NKG7*, *PRF1*) and immune checkpoint genes (e.g., *LAG3*, *PDCD1*, *LCK*) were significantly upregulated in T cells from the L group (*p* < 0.05, Wilcoxon rank-sum test, Supplementary Figure S7A, B). The scores of these functional gene sets were significantly correlated with CNV complexity score, implying the potential of subclonal complexity in predicting the activation status of T cells (Figure 5B).

We performed subclustering on T/NK cells, resulting in seven cell subpopulations: CD4_CCR7, CD4_IFNG+, CD4_FOXP3+_Treg, CD8_GZMK+, CD8+_TNSF9+, proliferative T cells, and NK cells (Figure 5C; Supplementary Figure S7C). The proliferative T cells were further annotated according to their similarity with other T subtypes and NK subtype (see methods, Figure 5D). Based on trajectory analysis of CD4^+^ and CD8^+^ T cells, we found that both CD4^+^ and CD8^+^ T cells from the L group tended to locate in the intermediate of the trajectory whereas those from the H group were primarily located at the terminal of trajectory (Figure 5E, F). Additionally, T cells from L group showed increased CytoTRACE score, indicating a heightened activation status of T cells in the L group (CD8+: *p* = 0.043, CD4+: *p* = 0.075, Wilcoxon rank-sum test, Figures 5E, F; Supplementary Figure S7D, E).

### Immune activation mediated by endothelial cell subtypes with varying subclonal complexity

**FIGURE 6:**
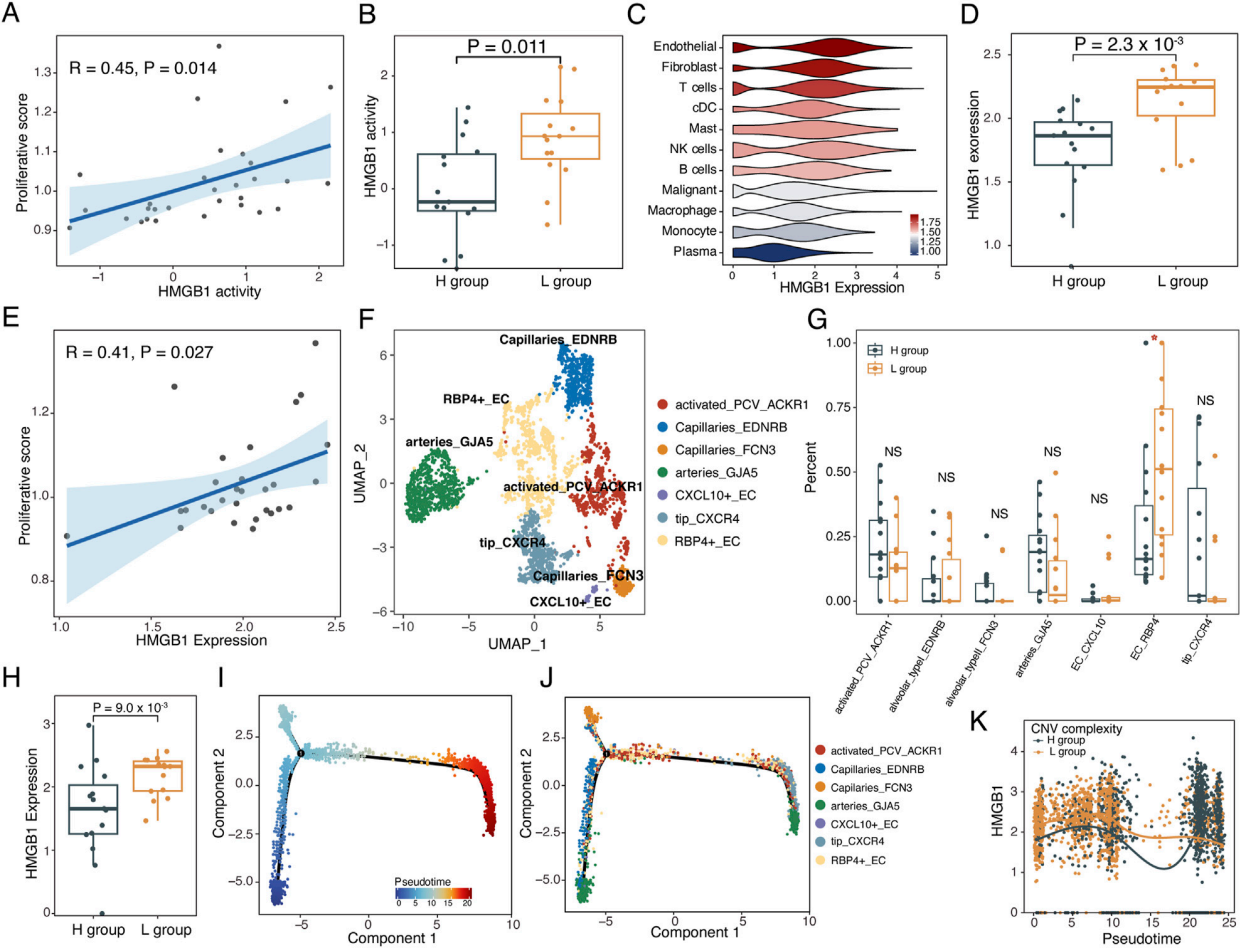
*HMGB1* drived the reprogramming of the TME. **(A)** Scatterplot showing the correlation between *HMGB1* activity and proliferative score of T cells. **(B)** Boxplot showing the *HMGB1* activity in L and H groups. Two-sided Wilcoxon rank-sum test. **(C)** Violin plot of expression levels of *HMGB1* in various cell subtypes, colored by the mean expression levels of *HMGB1* in cell subtypes. **(D)** Boxplot showing the expression levels of *HMGB1* in L and H groups. Two-sided Wilcoxon rank-sum test. **(E)** Scatterplot showing the correlation between the expression level of *HMGB1* and proliferative score of T cells. **(F)** UMAP plot of 7 endothelial cell subtypes from 30 samples. **(G)** Boxplots illustrating the fraction of endothelial subtypes in L and H groups. Two-sided Wilcoxon rank-sum test. *p < 0.05; **p < 0.01; ***p < 0.001; NS not significant. **(H)** Boxplot showing the expression of *HMGB1* from RBP4_EC in the H and L groups. Two-sided Wilcoxon rank-sum test. **(I–K)** Single-cell trajectory analysis of endothelial cells. **(I)** Cells were colored by pseudotime. **(J)** Cells were colored by cell subtypes. **(K)** The changes in expression levels of *HMGB1* along with the pseudotime.

To investigate the regulatory mechanisms underlying the different activation states of T cells, we estimated the signaling activities of 51 cytokines on T cells and their correlation with T cell proliferation using the CytoSig model (Jiang et al., 2021). The activity of the cytokine *HMGB1* showed a significant positive correlation with the proliferative score of T cells (*p* = 0.014, R = 0.45, Figure 6A). Moreover, *HMGB1* signaling activity was significantly higher in the L group compared to the H group (*p* = 0.011, Wilcoxon rank-sum test, Figure 6B). *HMGB1* was primarily expressed in endothelial and fibroblast cells (Figure 6C). However, the expression levels of *HMGB1* in endothelial cells, rather than fibroblast cells, were upregulated in the L group (*p* = 0.0023, Wilcoxon rank-sum test) and significantly correlated with T cell proliferation (Figures 6D, E; Supplementary Figure S8A). Additionally, the expression levels of *HMGB1* in endothelial cells was positively correlated with the activity scores of T cell exhaustion and TCR signaling pathway, and negatively correlated with the activity score of type II interferon response score (Supplementary Figure S8B).

We further dissected the subtypes of endothelial cells, identifying seven distinct endothelial cell subtypes (Figure 6F; Supplementary Figure S8C). The proportion of *RBP4* positive endothelial cells (RBP4+EC) was significantly higher in the L group compared to the H group (*p* < 0.05, Wilcoxon rank-sum test, Figure 6G). Moreover, *HMGB1* expression which was present only in RBP4+ ECs was significantly increased in the L group compared to the H group (*p* = 0.009, Wilcoxon rank-sum test, Figure 6H; Supplementary Figure S8D). Trajectory analysis demonstrated that RBP4+ECs persisted throughout the differentiation trajectory and showed highly expressed *HMGB1* in the L group along with the pseudotime (Figures 6I–K). Moreover, CytoTRACE analysis suggested that RBP4+ECs in the L group showed a less differentiated status (*p* = 0.0079, Wilcoxon rank-sum test, Supplementary Figure S8E–G).

**FIGURE 7:**
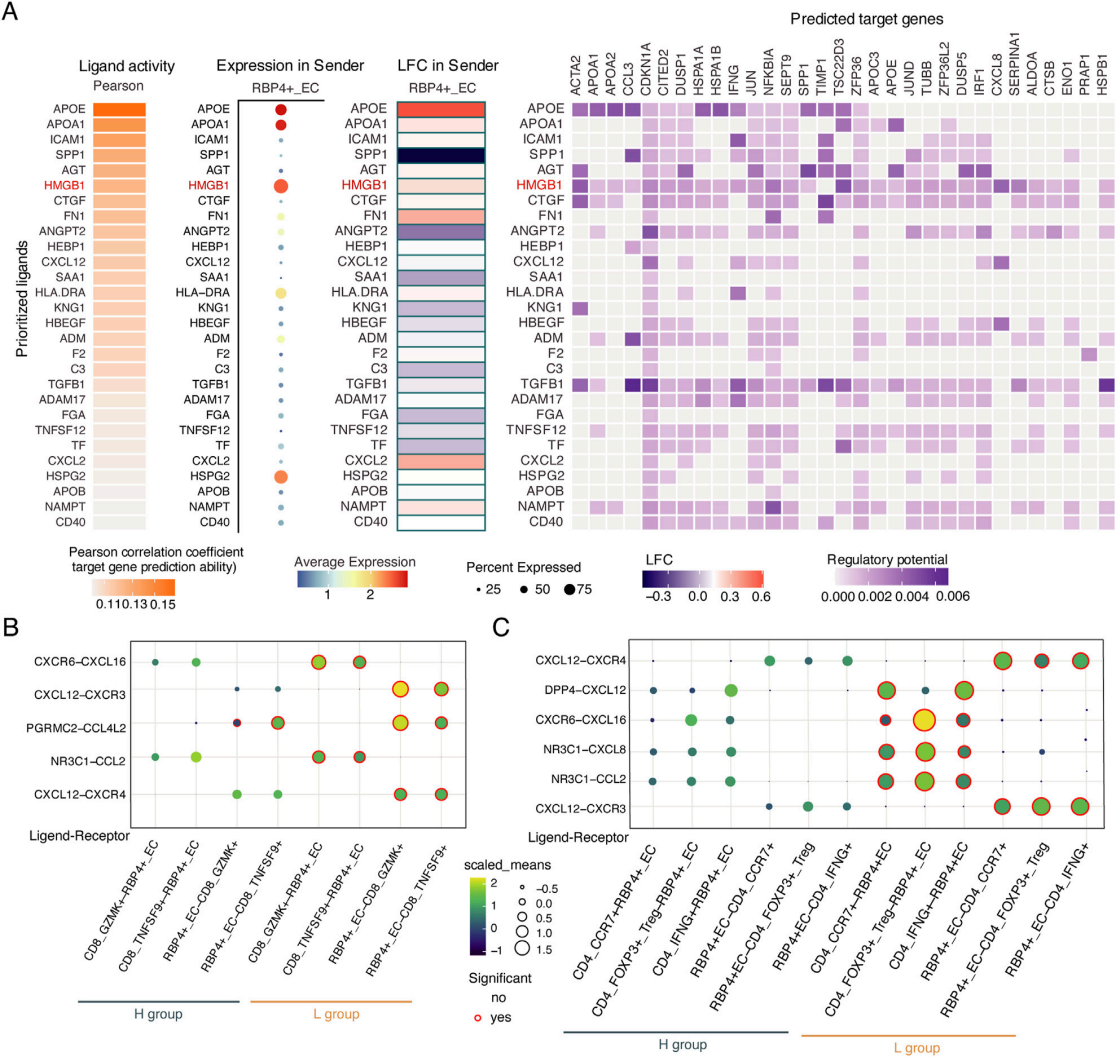
The interactions between RBP4+_EC and T cells. **(A)** Heatmaps showing potential ligands driving transcriptomic changes of target genes in T cells. **(B,C)** The chemokine-mediated ligand-receptor interactions between RBP4+_EC and T cells in L and H groups. **(B)** CD8^+^ T cells. **(C)** CD4^+^ T cells.

To further investigate the regulatory mechanisms of RBP4+ECs on T cell activation, we identified regulators from RBP4+ECs for T cell activation through Nichenet analysis (Browaeys et al., 2020) (Figure 7A). We found that RBP4+ECs regulated the expression of 29 target genes in T cells by upregulating *HMGB1* in the L group, including *IFNG*, which enhances tumor immune function and promotes T cell exhaustion (Benci et al., 2019), and *CCL3*, which facilitates dendritic cells (DC) recruitment for antigen presentation and T cell activation (Castellino et al., 2006). Interaction analysis between RBP4+ECs and T cells showed more and stronger chemokine-mediated interactions between RBP4+ECs and CD8+T in the L group including *CXCR6-CXCL16*, *NR3C1-CCL2*, *CXCL12-CXCR3*, *CXCL12-CXCR4*, and *PGRMC2-CCL4L2* (Figure 7B). Similar results were observed in the interactions between RPB4+ECs and CD4^+^ T cells (Figure 7C).

## Discussion

Tumor heterogeneity pervades across various cancers, profoundly influencing the survival of patients (Dagogo-Jack and Shaw, 2018). In this study, we observed sample-specific clustering patterns among malignant cells, whereas non-malignant cells exhibited clustering patterns predominantly defined by cell types. The correlation analysis of malignant cells revealed that intra-tumor heterogeneity was variable among samples and intra-tumor cells similarity was generally greater than that of inter-tumor cells. Previous research has identified complex transcriptional programs across diverse malignancies, including cell cycle, interferon response, partial epithelial-mesenchymal transition (pEMT), and complete EMT (cEMT) (Barkley et al., 2022). Here, we identified three meta-programs that were associated with distinct hallmark functions using NMF. Several strategies have been proposed to quantify the degree of tumor heterogeneity. For instance, Ma et al. (2019) employed PCA to project all malignant cells to the eigenvector space to derive PCs and calculated the averaged distance of malignant cells from the centroid (arithmetic mean of PCs of all malignant cells within the corresponding tumor) in principal component space. This averaged distance was used as a measure of heterogeneity for that particular sample. Guo et al. first calculated inter cell distances based on the Pearson correlation between any two malignant cells within a sample. The distance between cells was defined as D = 1- R, where R is the Pearson correlation coefficient between the CNV profiles of any two cells. Then, the metric of heterogeneity was defined as the median distance of distribution of pairwise cell distances (Guo et al., 2022). However, these methods implicitly involved the cell number in the calculation of heterogeneity, thus making them potentially influenced by the number of cells. Therefore, we proposed a new metric that avoided the involvement and interference of cell number and accurately quantified the complexity of subclonal structure without bias from cell numbers.

Data originating from glioblastoma (Patel et al., 2014; Neftel et al., 2019), oligodendroglioma (Tirosh et al., 2016), head and neck cancer (Puram et al., 2017), and melanoma (Rambow et al., 2018) consistently demonstrated a spectrum of differentiation within tumors, ranging from stem-like or progenitor states to fully differentiated cells, alongside a plethora of heterogeneous functional states in malignant cells, such as stress responses, interferon reactions, and hypoxia (Puram et al., 2017; Patel et al., 2014; Neftel et al., 2019; Moncada et al., 2020; Yu et al., 2022). We identified a total of 37 significantly different cancer functional states between low and high-complexity groups. The low-complexity group was significantly enriched in functional states associated with hypoxia, invasiveness, metastasis, stemness, apoptosis, and DNA repair, suggesting their potential roles in tumor progression and therapeutic resistance, driven by highly competitive dominant clones (Davis et al., 2017; Greaves and Maley, 2012). Furthermore, an investigation into lineage origins revealed that distinct compositions of cellular lineages in samples with varying subclonal complexity. Additionally, the TFs regulatory networks associated with the low and high-complexity groups reveal a correlation between subclonal complexity and survival outcomes in HCC.

Previous research has established that tumor heterogeneity is a critical cornerstone for reconstructing tumor evolution and driving the dynamics and diversity of the tumor microenvironment (Dentro et al., 2021). Genetic and epigenetic alterations collectively define the transcriptomic and phenotypic heterogeneity of malignant cells, leading directly or indirectly to the reprogramming of the TME (Flavahan et al., 2017; Vitale et al., 2021; Alizadeh et al., 2015). In this study, we observed markedly distinct interaction strength between malignant cells and other cell types within the microenvironment in high- versus low-complexity group. Specifically, malignant cells exhibited a substantially higher number of ligand-receptor interactions with myeloid cells, fibroblasts, and endothelial cells in low-complexity group compared to the high-complexity group. Notably, endothelial cells displayed the largest differences between the two groups, suggesting a potential role for endothelial cells in shaping the TME according to the complexity of subclonal structure. Our subsequent analysis revealed a remodeled immunological landscape in the low-complexity group, characterized by enhanced antigen-presenting capabilities of macrophages and heightened T cells immunoreactivity.

Cytokines play an important role in remodeling the tumor microenvironment (Binnewies et al., 2018). In this study, we identified *HMGB1,* a critical cytokine expressed by RBP4+ECs, which exerts immunostimulatory effects on T cells. Our results demonstrated that both the expression and signaling activity of *HMGB1* were significantly higher in the low complexity group compared to the high complexity group. A notable correlation was observed between the expression levels of *HMGB1* in endothelial cells and immune activity score of T cells. We further confirmed that RBP4+ECs had more frequent chemokine-mediated ligand-receptor interaction pairs with T-cells in the low complexity group. These findings collectively highlighted the intricate interplay between the subclonal complexity and the TME, particularly emphasizing the role of subclonal complexity in orchestrating immunological landscape. The increased interaction strength between RBP4+ECs and T cells and activation of T cells in the low complexity group, mediated by *HMGB1*, suggests a potential mechanism through which the cytokines can either promote or suppress antitumor immunity. Future studies should focus on elucidating the molecular mechanisms underlying the varying expression of *HMGB1* and its impact on T cells, potentially leading to the identification of novel biomarkers and therapeutic targets for cancer treatment.

## Data Availability

Publicly available datasets were analyzed in this study. This data can be found here: GSE156625 https://www.ncbi.nlm.nih.gov/geo/query/acc.cgi?acc=GSE156625 GSE149614 https://www.ncbi.nlm.nih.gov/geo/query/acc.cgi?acc=GSE149614 GSE151530 https://www.ncbi.nlm.nih.gov/geo/query/acc.cgi?acc=GSE151530 GSE189903 https://www.ncbi.nlm.nih.gov/geo/query/acc.cgi?acc=GSE189903.
