# Supplementary figures and images for: Single-cell transcriptomics reveals tumor microenvironment remodeling in hepatocellular carcinoma with varying tumor subclonal complexity
Source: Front Genet. 2024 Aug 29;15:1467682. doi: 10.3389/fgene.2024.1467682 (PMC11390501; doi:10.3389/fgene.2024.1467682)

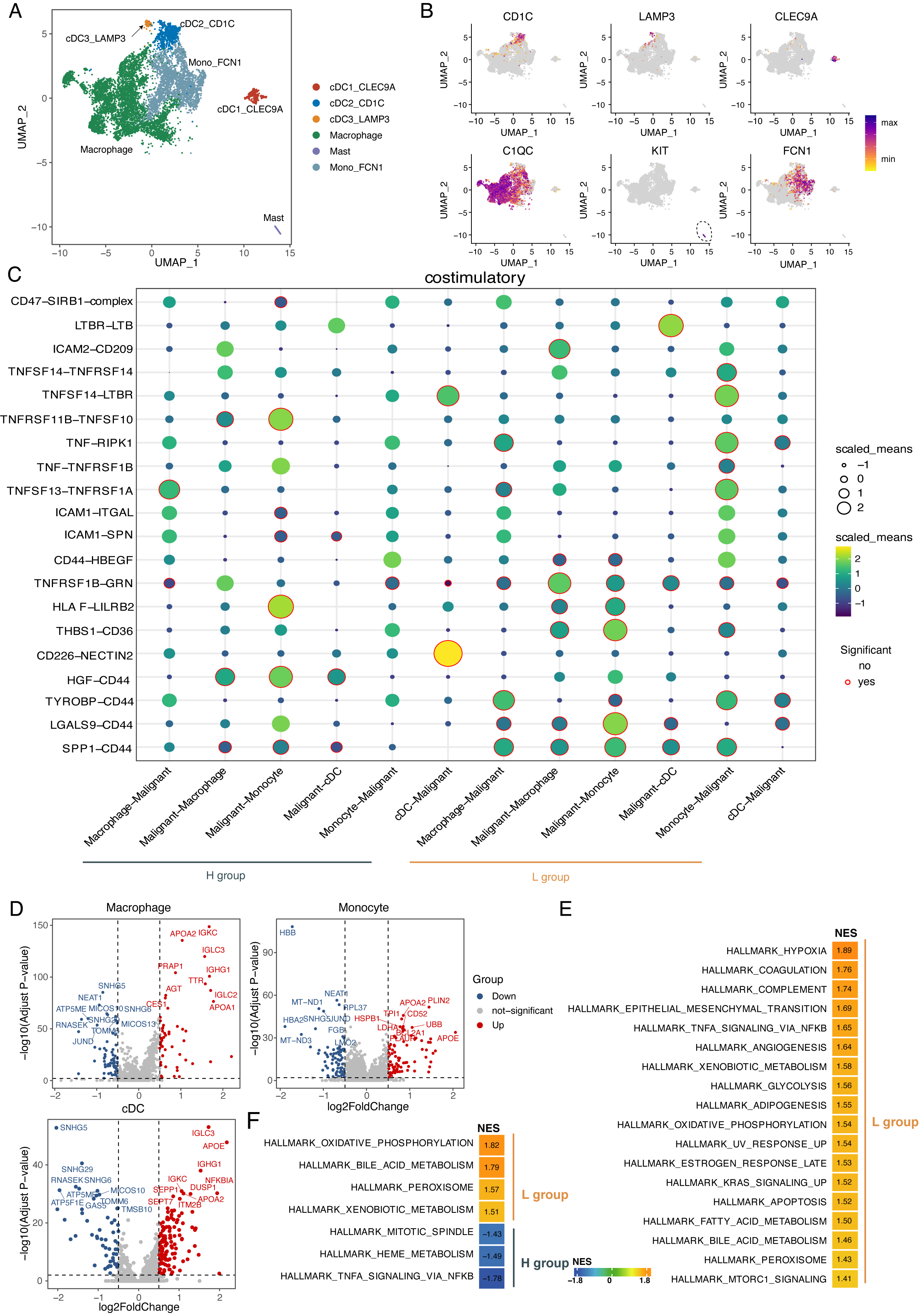

Supplement: Supplementary file 1 [file Image6.TIF]

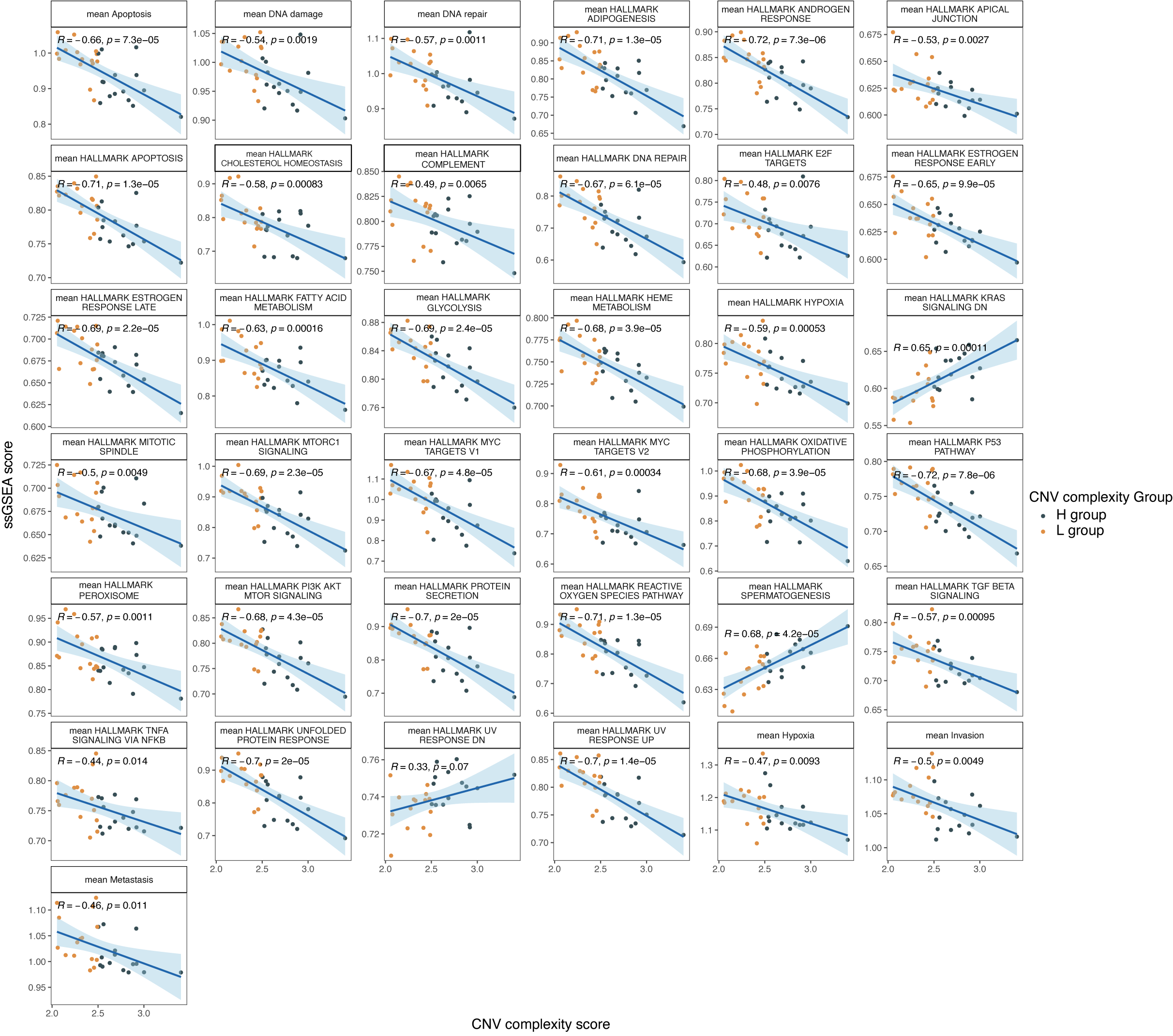

Supplement: Supplementary file 3 [file Image3.TIF]

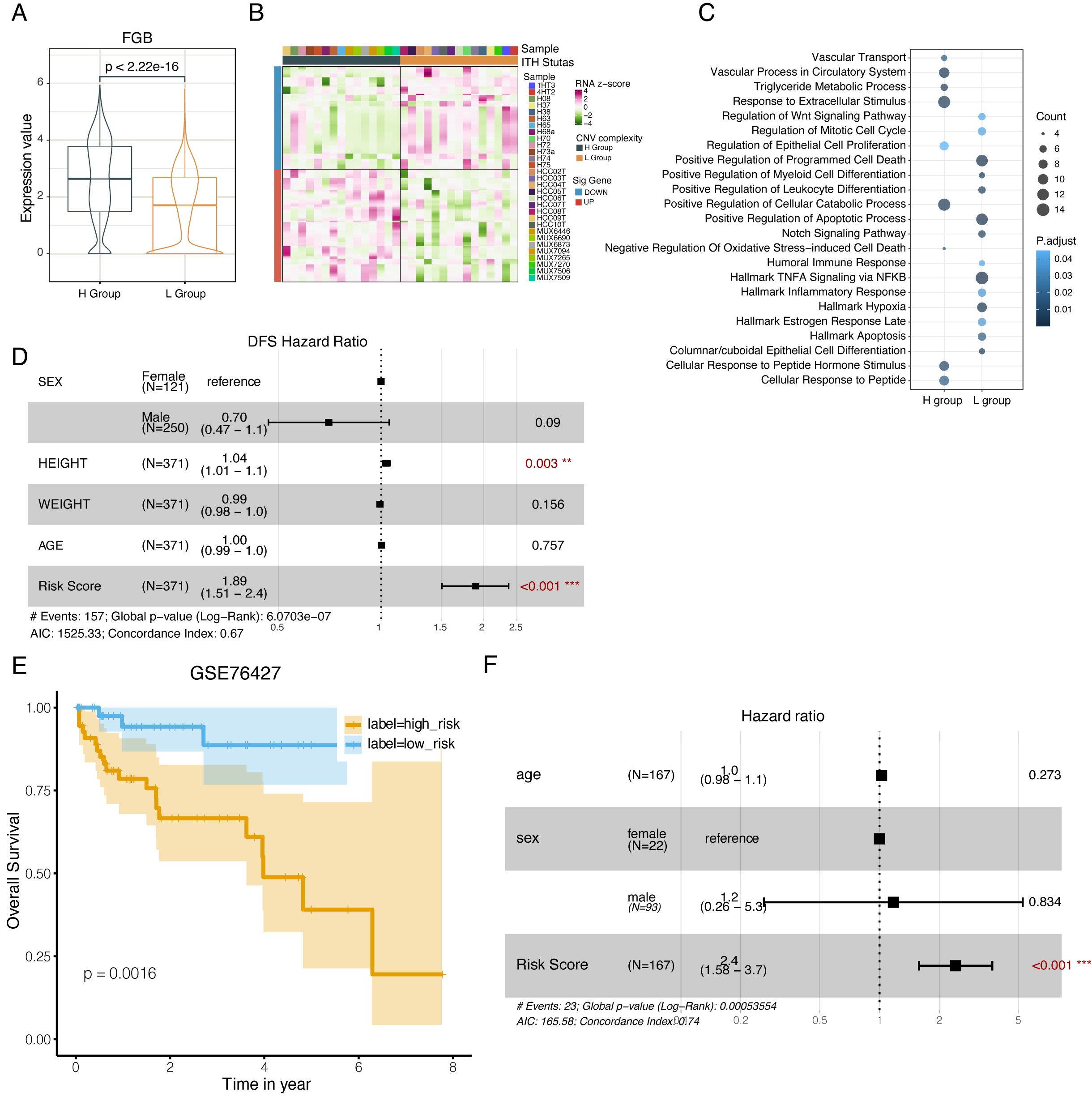

Supplement: Supplementary file 5 [file Image4.TIF]

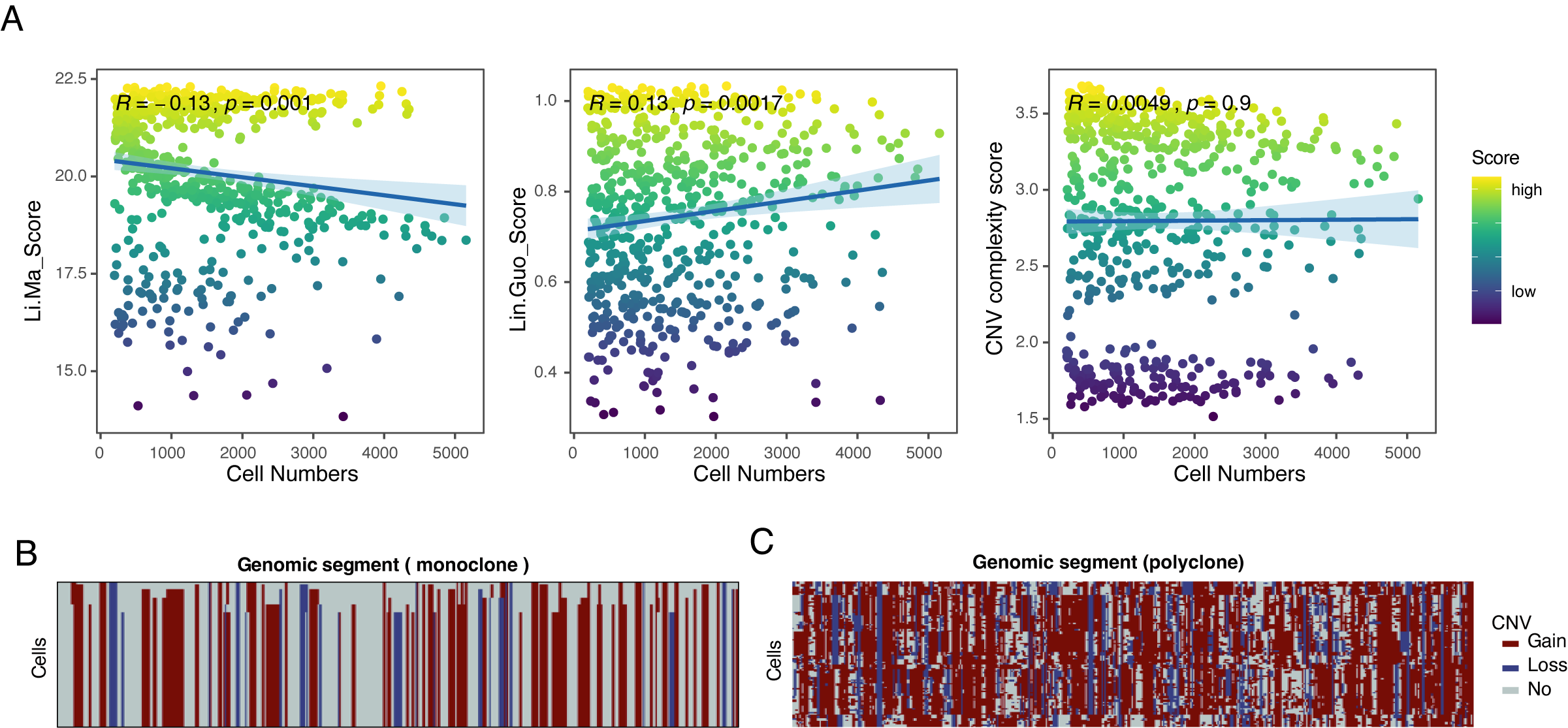

Supplement: Supplementary file 6 [file Image2.TIF]

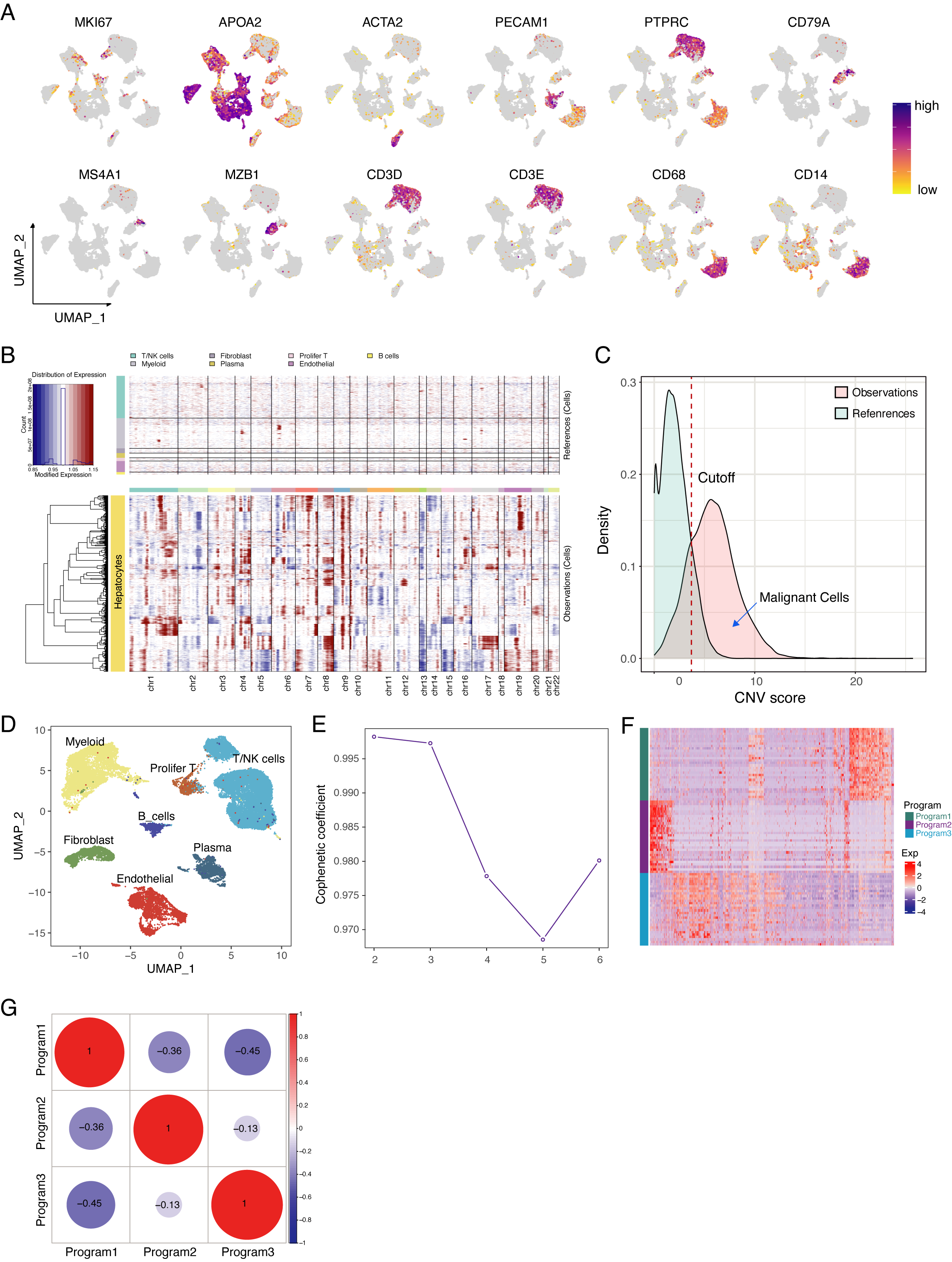

Supplement: Supplementary file 7 [file Image1.TIF]

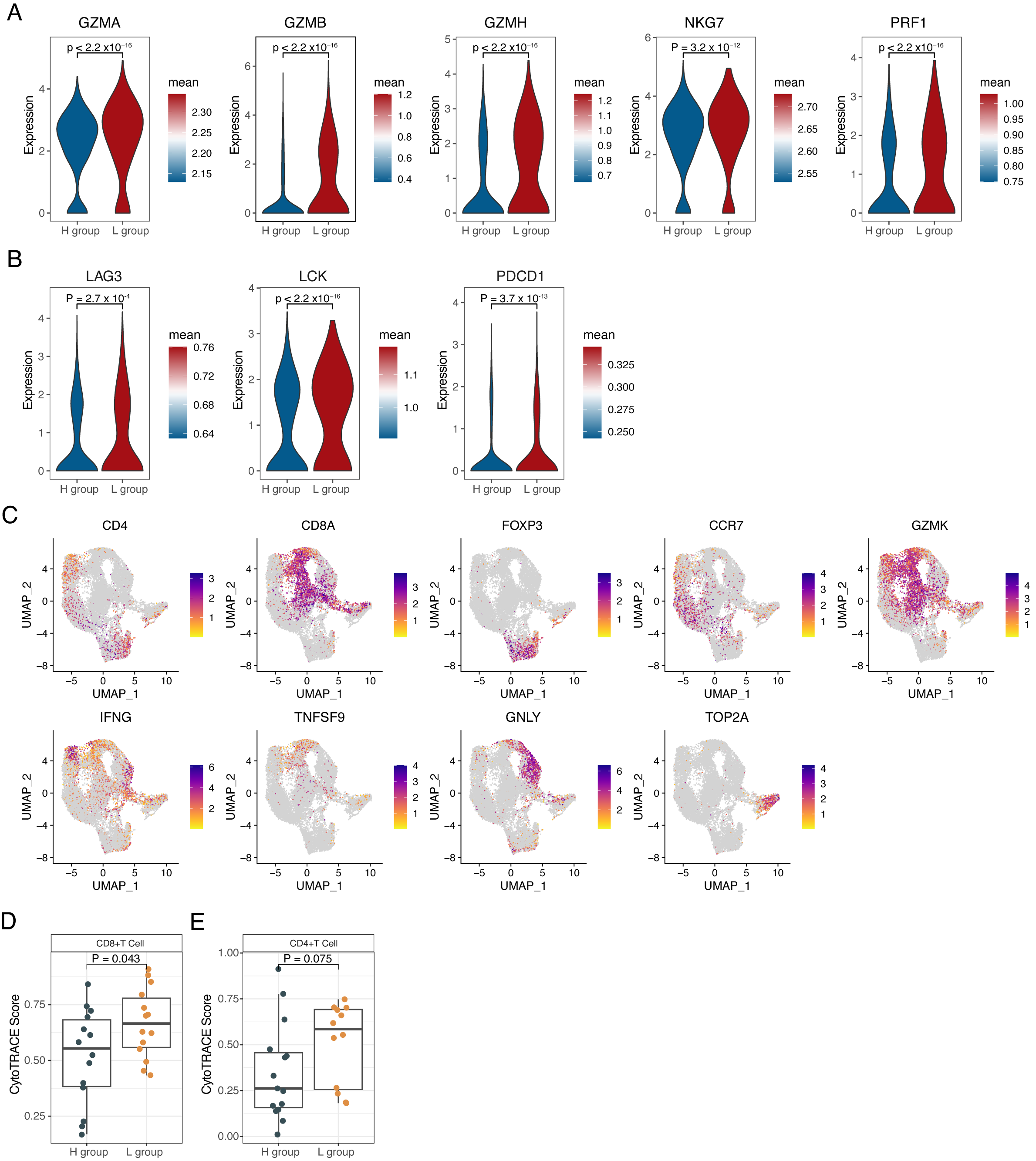

Supplement: Supplementary file 8 [file Image7.TIF]

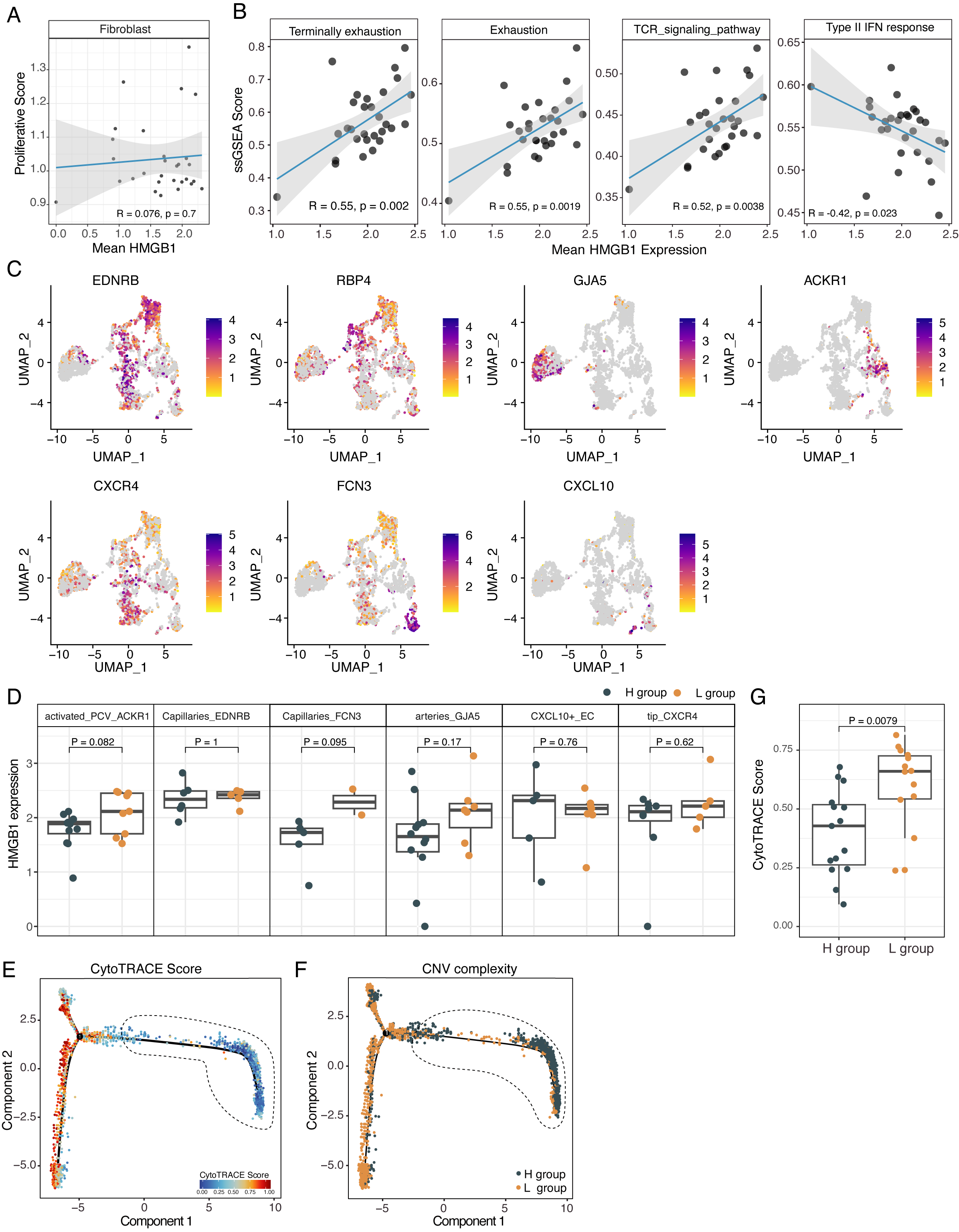

Supplement: Supplementary file 11 [file Image8.TIF]

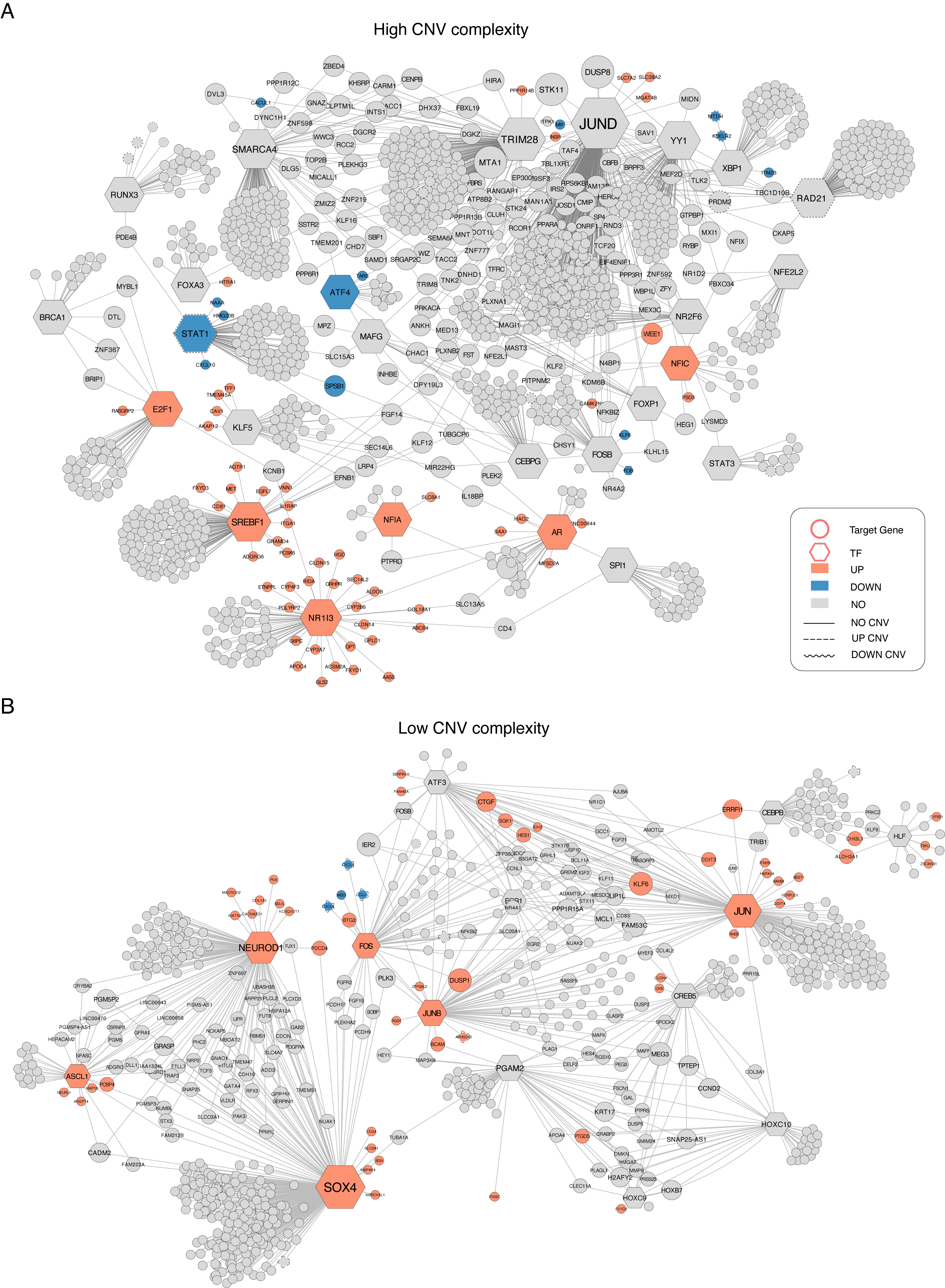

Supplement: Supplementary file 12 [file Image5.TIF]
