# Supplementary material for: Single-cell transcriptomics reveals tumor microenvironment remodeling in hepatocellular carcinoma with varying tumor subclonal complexity
Source: Front Genet. 2024 Aug 29;15:1467682. doi: 10.3389/fgene.2024.1467682 (PMC11390501; doi:10.3389/fgene.2024.1467682)
Supplement: Supplementary file 15 [file DataSheet1.docx]

**Single-cell transcriptomics reveals tumor microenvironment remodeling in hepatocellular carcinoma with varying tumor subclonal complexity**

Supplement Figure 1

Supplement Figure 2

Supplement Figure 3

Supplement Figure 4

Supplement Figure 5

Supplement Figure 6

Supplement Figure 7

Supplement Figure 8

**Supplementary Figure 1**. Tumor heterogeneity landscape of HCC.

1. Feature plot showing the marker genes expression for major cell type.
2. Heatmap of CNV signals normalized against the “reference” shown in the top panel for CNV changes by chromosome (columns) within individual cells (rows).
3. Distributions of CNV scores in hepatocytes (observation) and all non-malignant cells (reference). if the CNV score of a cell was greater than the cutoff, then it was a malignant cell.
4. UMAP plot of non-malignant cells from 30 samples. Cells were colored by cell type.
5. Line plots showing cophenetic measure changes in H72 sample.
6. Heatmaps of expression levels for genes of 3 gene programs in H72 sample.
7. Dot plots showing the Pearson correlation between pairs of gene programs in H72 sample.

**Supplementary Figure 2**. Construction and validation of the robust CNV complexity score.

(A) Scatterplot showing the correlation between the number of cells and Ma et al.’s score, Guo et al.’s score, CNV complexity score in simulation data. Each dot represented a sample.

(B-C) Generation of simulation data. (B) Copy number variations profiles for samples with low subclonal complexity (monoclone). (C) Copy number variations profiles for samples with high subclonal complexity (polyclone).

**Supplementary Figure 3**. Scatterplot showing the correlation between CNV complexity score and activity score of multiple cancer functional states. Each dot represented a sample.

**Supplementary Figure 4**. Characterization of the differences of malignant cells between the L and H groups.

1. Boxplot showing the expression levels of *FGB* from malignant cells in L and H groups. Two-sided Wilcoxon rank-sum test.
2. Heatmap showing the CNV signals of genes with statistically significant difference between L and H groups. Two-sided Wilcoxon rank-sum test.
3. Dotplot showing the related pathway enriched in L and H groups. The size of the dot represented the number of genes in the indicated pathway (the larger, the more genes included) and the color shade of the dot indicated the P value after FDR correction (the darker, the smaller adjusted P value).
4. Forest plot showing the HR (95% CI) for DFS. The multivariate Cox proportional hazard models. HR, hazard ratios; CI, confidence interval.
5. Kaplan-Meier plots of risk score on OS in validation dataset GSE76427.
6. Forest plot showing the HR (95% CI) for OS in validation dataset GSE76427.. The multivariate Cox proportional hazard models. HR, hazard ratios; CI, confidence interval.

**Supplementary Figure 5**. Construction of the subclonal complexity-specific TFs regulatory networks.

1. TF regulatory network of high CNV complexity.
2. TF regulatory network of low CNV complexity.

**Supplementary Figure 6**. Characterization of the functional roles of myeloid cells with different subclonal complexity.

1. UMAP plot of myeloid cells from 30 samples.
2. Feature plots showing the marker genes expression for major lineage of myeloid cells.
3. The costimulatory-mediated ligand-receptor interactions between malignant cells and myeloid cells in L and H groups.
4. Volcano plots showing the differentially expressed genes in macrophages, monocytes and cDCs with H group as control.

(E-F) Gene set enrichment analysis for (E) cDCs and (F) monocytes. Normalized enrichment score (NES) was used to display the enrichment of the related pathways. P.adjusted < 0.05 was considered to be significant.

**Supplementary Figure 7**. The immune activation of T cells in low CNV complexity.

(A-B) Violin plots showing the expression levels of (A) cytotoxicity-related genes and (B) immune checkpoint-related genes of T cells in the L and H groups. The color of a violin plot indicates the mean of the expression values.

(C) Feature plots showing the marker genes expression for major lineage of T cells.

(D-E) Boxplots showing the CytoTRACE score of (D) CD8+ T cells and (E) CD4+ T cells in the L and H groups. Two-sided Wilcoxon rank-sum test.

**Supplementary Figure 8**. *HMGB1* drived the reprogramming of the TME.

1. Scatterplot showing the correlation between expression levels of *HMGB1* from fibroblast and proliferative score of T cells.
2. Scatterplot showing the correlation between the expression levels of *HMGB1* from endothelial cell and activity score of exhaustion, terminally exhaustion, TCR signaling pathway and type II IFN response.
3. Feature plots for showing the marker genes expression for major lineage of endothelial cells.
4. Boxplots showing the *HMGB1* expression level of 6 endothelial subtypes in the L and H groups. Two-sided Wilcoxon rank-sum test.

(E-F) Single-cell trajectory analysis of endothelial cells. (E) Cells were colored by CytoTRACE score. (F) Cells were colored by subclonal complexity.

(G) Boxplot showing the CytoTRACE score of endothelial cells in the L and H groups. Two-sided Wilcoxon rank-sum test.
